# From Bivariate to Multivariate Analysis of Cytometric Data: Overview of Computational Methods and Their Application in Vaccination Studies

**DOI:** 10.3390/vaccines8010138

**Published:** 2020-03-20

**Authors:** Simone Lucchesi, Simone Furini, Donata Medaglini, Annalisa Ciabattini

**Affiliations:** 1 Laboratory of Molecular Microbiology and Biotechnology (LA.M.M.B.), Department of Medical Biotechnologies, University of Siena, 53100 Siena, Italy; lucchesi5@student.unisi.it (S.L.); donata.medaglini@unisi.it (D.M.); 2 Department of Medical Biotechnologies, University of Siena, 53100 Siena, Italy; simone.furini@unisi.it

**Keywords:** vaccination, multiparametric flow cytometry, mass cytometry, computational data analysis, automated analysis, machine learning

## Abstract

Flow and mass cytometry are used to quantify the expression of multiple extracellular or intracellular molecules on single cells, allowing the phenotypic and functional characterization of complex cell populations. Multiparametric flow cytometry is particularly suitable for deep analysis of immune responses after vaccination, as it allows to measure the frequency, the phenotype, and the functional features of antigen-specific cells. When many parameters are investigated simultaneously, it is not feasible to analyze all the possible bi-dimensional combinations of marker expression with classical manual analysis and the adoption of advanced automated tools to process and analyze high-dimensional data sets becomes necessary. In recent years, the development of many tools for the automated analysis of multiparametric cytometry data has been reported, with an increasing record of publications starting from 2014. However, the use of these tools has been preferentially restricted to bioinformaticians, while few of them are routinely employed by the biomedical community. Filling the gap between algorithms developers and final users is fundamental for exploiting the advantages of computational tools in the analysis of cytometry data. The potentialities of automated analyses range from the improvement of the data quality in the pre-processing steps up to the unbiased, data-driven examination of complex datasets using a variety of algorithms based on different approaches. In this review, an overview of the automated analysis pipeline is provided, spanning from the pre-processing phase to the automated population analysis. Analysis based on computational tools might overcame both the subjectivity of manual gating and the operator-biased exploration of expected populations. Examples of applications of automated tools that have successfully improved the characterization of different cell populations in vaccination studies are also presented.

## 1. Introduction

Flow cytometry allows to simultaneously quantify expression of extracellular and intracellular molecules targeted by dyes or monoclonal antibodies, as well as to measure multiple characteristics of a single cell such as size and granularity. This technology emerged as a powerful tool for detailed analysis of complex populations and several other factors have contributed to the success and widespread use of flow cytometry. These include the speed at which cells are analyzed, the high accuracy and resolution of the technology, and the low operating costs per sample. The more recent mass cytometry, or cytometry by time-of-flight mass spectrometry (CyTOF), is another technique for measuring the expression of more than 40 parameters on large number of cells. In mass cytometry, antibodies specific to markers of interest are conjugated to heavy-metal isotopes and used to stain a population of cells. Compared to mass cytometry, conventional flow cytometry is a non-destructive techniques which can be used to sort cells for further analyses and offers the highest throughput with tens of thousands of cells measured per second [1]. Considering the similarity of their outputs (files in Flow Cytometry Standard format), flow cytometry and mass cytometry share many analysis tools. While both techniques allow to interrogate the immune system at a previously unprecedented level, scientific progress depends on our ability to interpret these results. Classical analysis is performed by the operator, which manually explores the cells, and identifies cellular subsets by specific gates, that can be further analyzed for the expression of different markers combinations, thus providing a hierarchical analysis strategy. Manual analysis of cytometry data is a simple and intuitive one. However, it constitutes a big source of variability and it is time consuming when a large number of samples and markers are analyzed [2,3,4]. Moreover, experts are typically looking for specific and expected cell types, excluding other cells from the analysis [5]. Operator subjectivity occurs at the level of choosing the hierarchy in which parameter combinations have to be considered, as well as in the shape and boundary of each gate specified in the analysis. To overcome these limitations, novel computational techniques have been developed in recent years, and computational flow cytometry has become a novel discipline useful for providing a set of tools to analyze, visualize, and interpret large amounts of cell data in a more automated and unbiased way.

Comparative studies between traditional manual gating versus automated analysis have demonstrated that many of the available tools can efficiently achieve the same results produced by manual analysis [6,7] with the advantage of being operator-independent and able to identify also unexpected cell populations, that would be hardly identified with traditional methods. Supported by these positive results, in the last years automated analyses have been applied to the identification of different cell populations in pre-clinical and clinical studies in different fields such as immunology, vaccinology, cell biology, oncology, and hematology, contributing to a deeper understanding of biological processes. Since flow cytometry is a powerful technology for studying multiple immune function in response to vaccination, ranging from the phenotypic and functional characterization of cellular immune responses to antibody detection and functional assessment, the use of computational tools represents a powerful strategy for the interpretation of large datasets that can be instrumental to profile the vaccine immune response. For these reasons, immunologists should be aware of the potentiality of automated tools, which should not remain exclusive to computer-science experts 

In this review, a pipeline for the automated analysis of multiparametric cytometry data is described, focusing on both the pre-processing and analysis phases. The advantages/disadvantages, potentialities, and possible applications of the most commonly used algorithms currently available are described in order to make immunologists/vaccinologists aware of the added value of the computational analysis approach. Moreover, an overview of the impact of automated analysis in the knowledge of biological processes, especially in the vaccine field, is presented. 

## 2. Automated Cytometry Data Analysis Workflow

**Figure 1:**
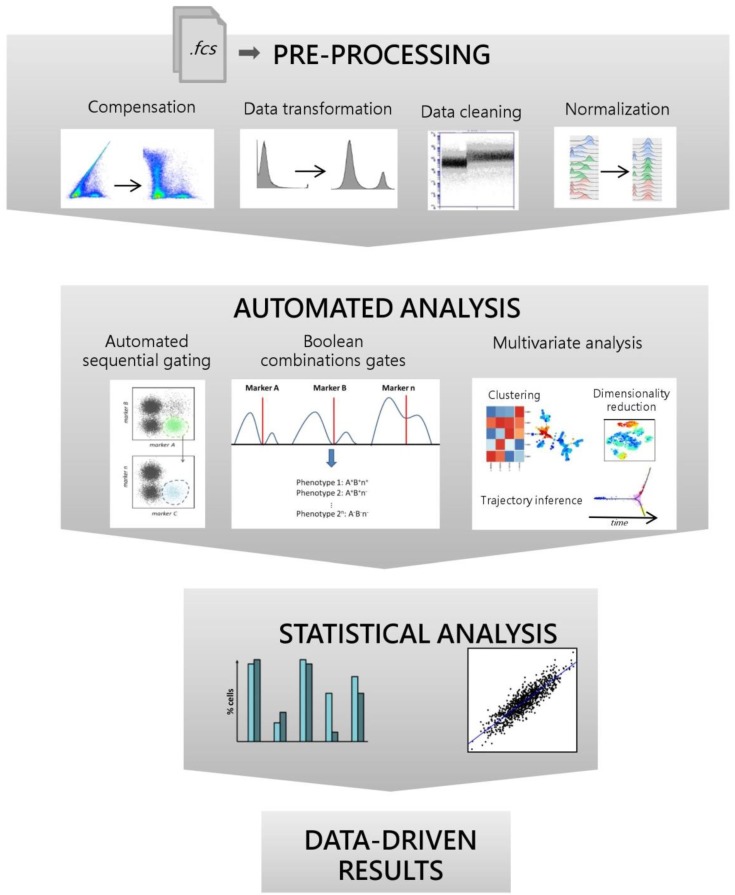
Automated cytometry data analysis workflow. Pipeline of the automated analysis steps of cytometric data. Starting from Flow Cytometry Standard (FCS) files, data are pre-processed to ensure reproducible and reliable results. Pre-processing phases include compensation (spectral overlap correction), data transformation (improvement of cell population visualization and automated cell types identification), data cleaning (removal of dead cells, debris, doublets, etc.), and normalizations (removal of batch effect between samples or balancing the contribution of each marker to the analysis). Pre-processed samples are analyzed with automated tools here classified as “Automated sequential gates”, “Boolean combinations gates”, and “Multivariate analysis” which include “Clustering algorithms”, “Dimensionality reduction methods”, and “Trajectory inference” techniques. Finally, statistical tests, correlation analysis and supervised machine learning techniques, such as regression and classification, can be applied to detect differences between experimental groups or to discover biomarkers.

The workflow for the analysis of cytometric data includes pre-processing, automated analysis with data-visualization, and result interpretation (Figure 1). Computational tools exist for each of these steps both as standalone software, FlowJo plugins, web services, and libraries for some of the most common programming languages. A high number of tools are available as packages for R, a highly popular language for statistical analyses, and stored on the Bioconductor repository, an open-source free software project for the analysis of high-throughput biomedical data [8] (Table 1).

The term computational cytometry is commonly referred to this vast arsenal of tools proposed for supporting the analysis of cytometry data. Different tools are characterized by a different degree of automation. At one side of the spectrum, there are tools that provide a complete unsupervised clustering of cell populations based on Machine Learning models, while at the other side, there are supervised tools that are specifically designed to assist in the manual analysis. In this review, the terms automated or computational analysis and automated tools will be used for indicating the computational analysis workflow and the different algorithms, respectively. The entire workflow of the computational analysis will be described step by step, starting from the pre-processing tools, through the different analysis phases, up to the result interpretation (Figure 1).

## 3. Data Pre-Processing

Cytometric data are usually provided in the form of FCS files. Before proceeding with further analyses, the raw-data included in FCS files needs to be pre-processed. Data pre-processing can be subdivided in four principal steps: (i) compensation; (ii) transformation; (iii) cleaning; and (iv) normalization (Figure 1).

Compensation. This step is necessary for adjusting the overlap between adjacent emission spectra of different fluorochromes. Compensation algorithms are included in most of the acquisition and analysis software packages that automatically perform this calculation. 

**Figure 2:**
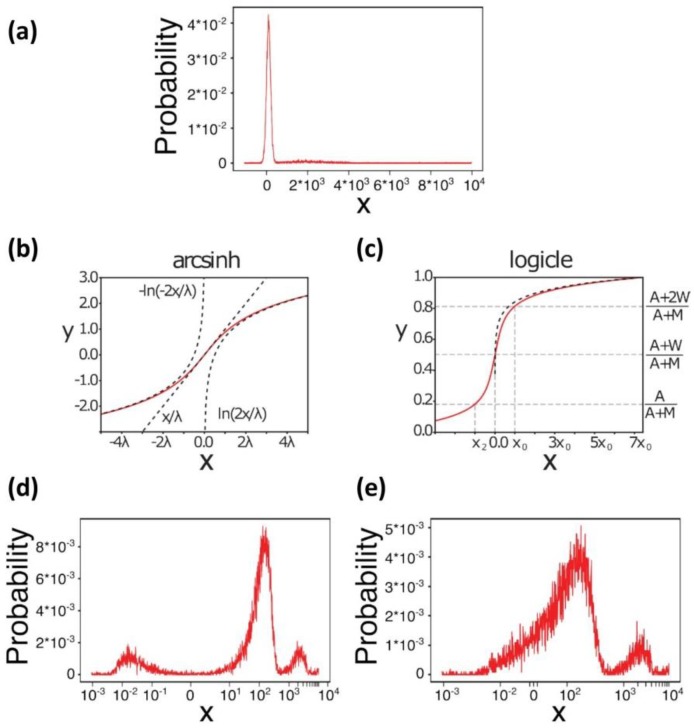
Data transformations. (**a**) A random data set was generated using two gaussian distributions, centered at 10^2^ (10000 cells) and 2*10^3^ (1000 cells), and with standard deviation equal to 10^2^ and 10^3^, respectively. The probability histogram is shown on a linear scale. (**b**) Arcsinh transformation. The parameter λ defines both the width of the linear region, and its slope. The transforming function is approximately linear for λ close to zero, while it approaches logarithmic transformations when x >> λ or when x << −λ. (**c**) Logicle transformation. The shape of the transforming function is defined by the parameters M, A, and W, which can be intuitively interpreted respectively as the number of decades, the number of negative decades, and the width of the linear region. (**d**) Probability histogram of the data in a transformed with the arcsinh function. (**e**) Probability histogram of the data in **a** transformed with the logicle function.

Data transformation. Data transformation is an important step in automated analysis, as well as in manual analysis, that facilitates cell population visualization and identification. Automated tools can be influenced by asymmetric cell populations, frequent outlier events, cell populations whose variance depend on their mean fluorescence intensity, and multiplicative errors in the fluorescence channels. Data transformation plays an important role in mitigating these effects [51]. Data from flow cytometry experiments usually range over 3-5 orders of magnitude. Thus, mapping, at least part of the data range, to a logarithmic scale is often required, both for efficient visualization and automatic analysis with Machine Learning algorithms. However, standard logarithmic transformations are rarely adopted in flow cytometry for two main reasons. Firstly, as a consequence of the compensation step, described in the previous section, input data might assume negative values, which obviously cannot be represented into a pure logarithmic scale. Moreover, a logarithmic scale would shrink the data range close to zero, which could hamper the identification of cell populations with low marker values. A common strategy to avoid these problems is to transform data using the inverse of the hyperbolic sine, as this function guarantees an almost linear transformation for values close to zero, while it approaches a logarithmic scale both for highly positive and highly negative values. A drawback of the hyperbolic sine transformation is that it uses a single parameter to control both the width of the linear regime and its slope. In order to remove this restraint between width and slope, which could prevent the identification of an optimal transforming function, it was proposed to use biexponential functions for data transformation (which can be considered as generalization of the hyperbolic sine), and in particular a special class of biexponential functions known as the *logicle* transformation. An advantage of using the *logicle* transformation is that the parameters of the transforming function can be more easily defined, and that they are linked to easily interpretable characteristics of the original data (Figure 2).

Data cleaning. Marginal events, debris, dead cells, and doublets should be removed, either manually or automatically, as well as outliers, in order to use high quality data as input in the analysis. Tools like FlowClean [11] and FlowAI [12] aim to automatically remove cells derived from anomalies in the acquisition. Removing low quality cells reduces noise in data sets and avoids false positive results or loss of rare populations [12].

Normalizations. The previous steps are required both for manual and automated analyses, while data normalization is explicitly required only for multivariate analyses. The first step of data normalization is to estimate batch effects, i.e. inter-sample variation. The flowStats package includes two functions (warpSet and gaussNorm) to normalize the data on the base of high-density region landmarks for individual flow channels [10], while in mass cytometry normalization of batch effect can be performed with the packages CATALYST (Cytometry dATa anALYSis Tools) or CytoNorm [14,52,53].

In the second step of data normalization, the expression values of separate markers are modified so that different makers have similar expression ranges. This is needed as many clustering and dimensionality reduction algorithms compute the distance between cells or identify dense cell areas in multidimensional space, and these analyses would be hampered by the presence of highly different ranges among the various markers. To balance their contribution, each marker in the data set is normalized (normalization between markers, also referred to as scaling), employing the z-score or min-max normalization methods [32].

## 4. Automated Data Analysis

After pre-processing, the next phase in the automated analysis of cytometric data is the discovery and quantification of different cell populations. The main advantages and limits of different strategies are described below.

### 4.1. Automated Sequential Gating

Automated tools for sequential gating automatically compute gates around cell populations in bi-dimensional plots, overcoming the operator subjectivity due to manual drawing. Instead, the sequence of the markers analyzed is still defined by the operator. OpenCyto [16] and FlowDensity [15] R packages, as well as the standalone executable software AutoGate [17], assist the operator in the definition of mono- and bi-dimensional gates, by using methods for boundary definition based on density estimation techniques. The main advantage of the automated sequential gating approach is represented by the automated identification of the cell population in the bi-dimensional scatter plots, overcoming the limitations linked to the manual drawing of gate boundaries, thus improving reproducibility. An evolution of these algorithms is represented by tools that automatically identify the gating strategy. Cytometree, implemented as R package, aims to construct a binary tree in which the nodes represent gates and the binary tree represents the best mono-dimensional sequential gating strategy used to identify the cellular sub-populations [18]. The AutoGate software has been recently implemented with the Exhaustive Projection Pursuit (EPP) clustering approach which automatically detects the best two-dimensional gating strategy to identify the cellular sub-populations [19]. Automated detection of the gating strategy allows to analyze all cells in the dataset, without discarding cells from the analysis, as occurs when the gating strategy is user dependent.

### 4.2. Boolean Combination Gates

Boolean combination gates analyze all the possible combination of marker expressions, overcoming the issue of selecting pairs of parameters and the hierarchy that characterize manual analysis. This approach is fast, capable of considering all cells in a dataset, and it allows to compare different phenotypes across samples. The main limitations of Boolean gates regard the visualization of the results when the number of parameters increase and the difficulty of separating populations in the mono-dimensional gating step (Supplementary Figure S1). 

A tool for performing Boolean combination gating is flowType, which is an R package available on Bioconductor repository, that automatically bisects cells in positive and negative for each analyzed marker [20], and it provides as output all possible phenotype combinations, including parent populations. Although the high number of possible phenotypes hampers the visualization of the results, this approach is particularly suitable for biomarker identification, since it explores the dataset considering all the possible marker combinations. To this aim, FloReMi and RchyOptimyx packages might be used to better interpret flowType results [21,22]. In addition to R packages, Boolean combination gates are also available as a FlowJo tool.

### 4.3. Multivariate Approach

Algorithms based on clustering, dimensionality reduction, and trajectory inference fully switch from the univariate/bivariate analysis to a multivariate approach. These tools consider the distribution of all markers simultaneously in the whole dataset, overcoming many of the manual gating limitations. 

#### 4.3.1. Clustering

Clustering based approaches identify and separate cells with similar marker profiles into cell clusters. This is the only multivariate approach which allows to quantify cell subsets in different samples and to perform comparative analysis between different experimental groups (e.g. stimulated samples versus control). The clustering tools can be classified on the basis of the kind of algorithm used for diving the cells into separate populations. SPADE (spanning tree progression of density normalized events) [54] and Citrus [26] are based on hierarchical clustering algorithms. The popular K-means, in which events are iteratively assigned to *k* clusters, is the algorithm on which flowMeans is based [23]. In PhenoGraph, cells are connected by weighted edges, where sets of highly interconnected cells represent phenotypically similar cell (or “communities”) that can be partitioned in clusters using similar community-detection algorithms used for the analysis of social networks [35]. Algorithms such as flowClust [29], immunoClust [30], SWIFT [31], and HDPGMM [25] are model-based techniques and assume each cell type can be modelled as a multivariate statistical distribution. Other tools are built upon density-based algorithm, such as FLOCK (FLOw Clustering without K) [32], X-shift [28], flowPeaks [33], and ClusterX [34], in which more dense regions are identified and used as cluster centers.

An evaluation of the performance of automated gating techniques can be found in Weber and Robinson [55], where different algorithms are compared with manual gating. In the Weber and Robinson benchmark, FlowSOM [27] has emerged as one of the algorithms with highest performance for the automated identification of cell populations (measures as F-score with respect to manual gating results), being at the same time one of the fastest ones. FlowSOM has been recently included in FlowJo as a plugin.

Visualization of clustering results is an important step to appropriately interpret the results and many tools include visualization implementation. Histograms and dot plots are used to display marker distribution in a cluster comparing with a parental population. Other approaches aim to visualize inter-cluster relationship, showing clusters centroids, cluster median values or frequency of positive cells with minimum spanning three (MST), heatmaps, scaffold map, and dimensionality reduction techniques [27,54,55,56,57].

#### 4.3.2. Dimensionality Reduction

Dimensionality reduction techniques aim to map high-dimensional data into a lower-dimensional space by losing as little information as possible. In the field of cytometry, dimensionality reduction is usually adopted to easily visualize the data, generally in two- or three-dimensional plots. These low-dimensional plots provide a straightforward visualization of the structure of multidimensional data, maintaining the information of data at a single-cell level, which instead is lost in clustering analyses. Principal component analysis (PCA) is a widely used method for reducing the dimensionality of multivariate data by linearly mapping the original variables into a low number of principal components (PCs). The resulting PCs represent a new set of variables oriented along the direction of maximum variance in the original dataset. Since PCA performs linear transformations to reduce dimensionality, it might be not optimal for reducing the number of dimensions in biological systems, where nonlinear relationships are common. This shortcoming might produce artefacts in low-dimensional space, with two points close in the low-dimensional space but not in the original multidimensional space. In cytometry, one of the most commonly used dimensionally reduction technique, that overcomes the limitation of linear transformations inherent in PCA, is the t-distributed stochastic neighbor embedding (t-SNE) algorithm [36], a tool available in R and in FlowJo. This method aims to map points from the high-dimensional space to the low-dimensional map by minimizing the difference in all pairwise similarities. Two of the most used tools for analyzing cytometric data, based on the t-SNE algorithm, are viSNE [38] and Automatic Classification of Cellular Expression by Nonlinear Stochastic Embedding (ACCENSE) [37]. 

The main drawback of t-SNE is the high computational cost of the algorithm, with the consequence that usually the low-dimensional maps are built using a limited number of cells obtained with a down-sampling of the original data. Moreover, it is important to remark that the algorithm includes a series of stochastic steps and consequently different analyses will give slightly different results. Recently, new dimensionality reduction tools such as EmbedSOM [39], diffusion maps [58,59], Fit-SNE [42], and UMAP (Uniform Manifold Approximation and Projection) [40] have been developed and applied to single-cell data to overcame t-SNE limitations. Dimensionality reduction is purely a visualization tool and does not allow the exact quantification of the identified population that requires a subsequent step. In flowJo, a manual gating analysis can be performed on dimensional-reduced t-SNE map, while other tools such as ACCENSE [37] perform an automated gating on t-SNE map.

#### 4.3.3. Trajectory Inference

The last and most recently developed approach for analyzing single-cell dynamic processes are the trajectory inference methods. This approach aims to model the cell development and the transitions between different cell states by following marker expression gradients in the multi-dimensional data set. 

Trajectory inference makes a step forward compared to clustering and dimensionality reduction algorithms, allowing at a single-cell level the unbiased study of cell processes such as the cell cycle, cell differentiation and cell activation. Starting from a mixture of different cells, these algorithms reconstruct the development stages that cells are following, ideally sorting the immature cells first, followed by the transitional stages, and finally the mature cells. With multivariate algorithms, and in particular trajectory inference methods, the multicolor panel design is crucial, to ensure that all relevant transitional states can be detected [60]. While different trajectory inference approaches have been developed for single-cell transcriptomic application [61,62,63,64,65], a limited number of methods have been applied to cytometry analysis. Wanderlust detects linear transition, starting from a user-defined starting cell and subsequently ordering the rest of the cells [43], on the other hand, Wishbone, Monocle, and PHATE are able also to detect a bifurcation in the trajectory, enabling to characterize different cell lines that are difficult to identify within a linear model [44,45,46]. The interest in the trajectory inference field is growing rapidly, with a rising number of tools developed each month [63]. Their application in studying cell differentiation and development is currently limited to single-cell transcriptomics data, but it is likely that in the near future many of these tools could also be used to analyze data from flow and mass cytometry.

#### 4.3.4. Multivariate Analysis Settings

Multivariate analysis bypasses the need to make choices that could influence the results, such as the definition of gates or the sequence of analyzed markers. However, the selection of optimal parameters by the operator still plays a key role, and sometimes the analysis has to be repeated multiple times in order to identify the best settings [66]. A crucial point in multivariate analysis is the number of target cell populations that is required as input parameter in many tools such as FlowMeans, FlowSOM, or SPADE. In the definition of the number of clusters, two conflicting requirements need to be taken into account. With a high number of clusters, the clusters are more homogenous, and it is more likely to identify rare populations, but visualization and interpretation of the results is highly complicated by over-fragmentation and noise. On the other hand, a low number of clusters makes visualization and interpretation easier, but it increases the likelihood of missing interesting cell populations. A common method for estimating the optimal number of clusters is the “elbow-method”, in which the sum of the square distances of all the samples from the corresponding cluster centers (cost function) is plotted as a function of the number of clusters. Nevertheless, it is not always easy to identify the optimal number of clusters, therefore, it is advisable to set the number of clusters slightly higher than expected populations in order to ensure that the relevant cell types can be found [60].

Algorithms that do not directly require the number of clusters could still include parameters that affect the number of populations. For example, FLOCK, a density-based clustering algorithms, has two main parameters (the number of bins and the density threshold) that influence the estimated density and, indirectly, the number of resulting cell populations [32]. In t-SNE, perplexity is a parameter that influences the similarity measure. Roughly, with a low perplexity, the algorithm considers as similar only the nearest cell, resulting in an over-fragmentation of the populations; while, with a high perplexity, all cells are considered to have the same similarity, resulting in random distributed points on the map. Typical perplexity value are between 5 and 50 [36] and to get the most from t-SNE, it is recommended to analyze multiple plots with different perplexities. Another important choice is the selection of the parameters (markers) to include in the analysis. The so-called “curse of dimensionality” affects multivariate analyses when a high number of variables are considered in once. It was suggested that the curse of dimensionality could also affect multivariate analysis of cytometric data [67]. However, comparative studies by the FlowCAP project have shown that many of multivariate tools have reached a level of maturity that matches, or even surpasses, the results produced by human experts [6,55,68]. Nevertheless, it is recommendable to choose the more appropriate markers to include in the analysis in order to reduce dimensionality, complexity, and noise of datasets (e.g.: removing from the analysis markers that show only negative population). 

## 5. Interpretation of the Results

The final phase in an automated analysis pipeline is the interpretation of the data-driven results. Generally, the cell populations identified have to be compared among different experimental groups. In manual analysis, statistical tests such as Mann–Whitney or Kruskal–Wallis are generally performed to identify populations with statistically relevant differences between experimental groups. When used in automated analysis, the use of multiple tests correction becomes necessary, such as Benjamini–Hochberg or Bonferroni, since many statistical comparisons are performed, increasing the probability that a type I error (false positive error) occurs. 

Correlation test or supervised Machine Learning methods, such as multivariate regression and classification, can also be used to identify a signatures that correlates with an external variable [6]. Multivariate regression is used to model an association with a continuous outcome variable, while classification methods can be used to identify links with a categorical clinical outcome of interest, such as a pathology. Once trained, these machine learning methods can be used to make predictions about new samples, where the output variables, being continuous or categorical, is unknown. Some packages, such as Cytrus and FlowSOM, includes possible statistical tests to be applied down-stream to the clustering analysis. 

## 6. Impact of Automated Analysis in the Knowledge of Biological Processes

**Figure 3:**
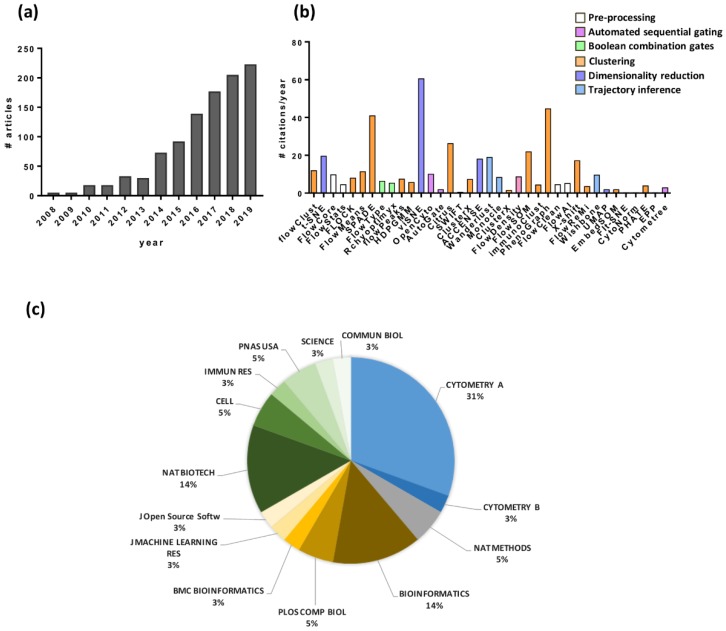
Analysis of the impact of automated tools on the scientific literature. (**a**) Numbers of publications per year, from 2008 to December 2019, on automated analyses of cytometry data. The query was performed with Web of Science starting from the articles reported in Table 1, selecting all the citing articles (7613), and refining the search for “cytometry” (1018 articles). (**b**) Numbers of citations each year of the reference articles of each automated tool reported in Table 1 (x axis); bars were colored according to the tool function. (**c**) Pie chart representation of the percentage of journals chosen for the publication of the reference article of each automated tool reported in Table 1.

Increasing numbers of automated analyses of multidimensional cytometry data have been published in the last years, as reported in Figure 3a. The analysis has been performed using Web of Science, starting from the articles describing the automated tools reported in Table 1, then selecting all the citing articles (7613), and refining the search for “cytometry” (1018 articles). The time course analysis shows a rising trend of publication starting from 2008, the year of publication of t-SNE [36] and flowClust [29], up to date, with a stronger increase in the 2014–2019 period. In these years, three special issues of Cytometry Part A, the journal specialized in quantitative single-cell analysis by cytometry techniques, have been entirely dedicated to the computational analysis of flow cytometry data. Two of them were built around the “Flow Cytometry: Critical Assessment of Population Identification Methods (FlowCAP)” project [69], aimed at advancing the development of computational methods for the identification of cell populations of interest in flow cytometry data, under the direction of an open consortium of immunologists, bioinformaticians, statisticians, and clinical scientists [70,71]. The third one, “Machine learning for single cell data”, is a special issue focused on the development and comparative analysis of machine learning methods and their application to single cell data, planned to be published in February 2020.

Articles reported in Table 1 are technical reports on software/methods development, where test datasets have been employed for evaluating their power or comparing the performance of available tools. The analysis shows that vi-SNE and ACCENSE, implementations of t-SNE, are the tools most cited for dimensionality reduction analysis, while Phenograph, SPADE, Citrus, FlowSOM, and X-shift for clustering approaches (Figure 3b). For the pre-processing step, FlowCore has the highest number of citations, since it offers essential function such as compensation and transformation of data, while new tools, such as FlowClean and FlowAI, recently published (2016) are aimed at data refinement, such as elimination of the outliers and anomalies during acquisition. Most of these tools are available in the R platform, and their use has been partially limited to bioinformaticians and researcher with programming expertise, even though they are available as open-access software. Indeed, analyzing the category of the journals selected for publication (Figure 3c), the majority (about 70%) are specialized in cytometry, methods and computer science, while only about 20% are in multidisciplinary and less than 10% immunology/life science journals.

An effort made to simplify the use of some automated tools has been the development of software with user-friendly interfaces an plug-ins capable of extending the functionality of the FlowJo software. This strategy has the advantages of combining the use of one of the most popular software for flow cytometry with automated analysis, thus helping the researchers to approach to the computational analysis of multiparametric data. 

Since comparative studies between manual versus automated analysis have demonstrated that many of the available tools can efficiently achieve the same results produced by manual gating [6,7], automated analysis have been applied, in recent years, for the identification of different cell populations in biomedical research and clinical diagnostic analysis [72]. One of the first computational approaches applied to clinical data analysis was conducted for identifying immunological correlations of HIV protection. Automated analysis was applied to a dataset derived from a large retrospective study of individuals at the early stage of HIV infection, and allowed to identify three T-cell subsets whose frequency during early infection had a statistically significant relationship with clinical progression to AIDS [20]. In the field of hematologic malignancies, successful application of computational methodologies have also been reported for acute lymphoblastic leukemia (AML) aimed at improving the discrimination between leukemic and normal cells [73,74], identifying B cell precursor as predictors of disease relapse [75], monitoring the minimal residual disease [76,77], evaluating the disease progression [78], or characterizing the immune alterations in AML patients [79]. Moreover, computational methods have been used on existing clinical flow cytometric data to improve diagnostic accuracy to distinguish mantle cell lymphoma from small lymphocytic lymphoma [80], or discriminate various subpopulations of blood cells in the context of B-chronic lymphocytic leukemia [81]. Complex data sets generated by multi-parametric flow cytometry have been analyzed with automatic tools for characterizing myeloid and lymphoid cells in steady state [82,83,84], during the differentiation process [85,86], and in pathological conditions [87,88,89,90,91,92,93].

The automated analysis approach is therefore a powerful tool for unambiguous and unbiased characterization of cells, their subpopulations, functions, and roles in physiological and pathological conditions, applicable both in biomedical research and clinical diagnostic analysis [72,94]. 

## 7. Flow Cytometry in Vaccine Studies and the Advantages of Computational Analysis

Flow cytometry is a powerful technology for the characterization of multiple immune functions in response to vaccination, and both humoral and cellular components can be measured and characterized by flow cytometry-based assays. Multiparametric flow cytometry can be particularly suitable for the deep characterization of cellular immune responses, allowing to measure the phenotype and the functional features of rare cells, such as antigen-specific cells. The study of the CD4+ T cell activation and their effector function is fundamental in the characterization of immune responses to vaccination [95]. T helper cells are indeed closely related with long-term humoral immunity and modulate the functions of macrophages and CD8+ cytotoxic T cells through cytokines secretion, thus playing a central role in mediating vaccine immune responses [96]. Through flow cytometry, it is possible to directly and specifically identify antigen-specific T cells, using the major histocompatibility complex (MHC) tetramer staining technology [97,98], a procedure that has been used for characterizing antigen-specific T cell responses both in pre-clinical and clinical studies [99,100,101]. Furthermore, the combination of tetramer-staining with intracellular cytokine detection allows to assess, at single-cell level, the polyfunctional activity of antigen-specific T cells [102,103]. These procedures can be applied to better understand the complex functional profile of CD8+ and CD4+ T cell responses upon vaccination or infection. 

Multiparametric flow cytometry can be particularly suitable also for characterizing polyclonal antibody responses elicited by vaccines, through a set of antibody-detection or cell-based functionality assays that can allow to identify humoral features that correlate with protection [104]. Antibody Fc-mediated mechanisms, such as cellular cytotoxicity, phagocytosis, direct pathogen killing, and modulation/stimulation of innate and adaptive immunity, can contribute, beyond neutralization, to confer protection against many pathogens. These mechanisms can be measured trough a range of different flow cytometry-based functional assays, that integrated with biophysical assays through Machine Learning methods, can contribute to profile the polyclonal antibody response and to identify immunological correlates and mechanism of humoral protection [104,105]. A complementary approach to the antibody response characterization is the study of the B cell response to vaccination, in which the production of plasma cells and memory B cells can be deeply analyzed by flow cytometry and its development and dissemination can be tracked between lymphoid organs and blood. 

Automated analysis of cytometry data represents a powerful tool for the interpretation of large datasets in an unbiased way, that can be instrumental to profile the vaccine immune response. This analysis might unmask the detection of specific phenotypes/effector cells, that could be hardly detected with the manual analysis, and identify particular cell types (biomarkers) that can be specifically induced by tested vaccine formulations. Automated analysis has become particularly necessary as the size of marker panels has increased and consequently the number of cell populations identified by the combination of different markers has exponentially raised. Thanks to the computational approach, it is possible not only to identify cell populations according to the expression of two or three well-known specific surface markers, but also to distinguish different subsets within a population, based on combination of the other surface molecules expression. These subsets can be cells at intermediate stages of differentiation, or novel unexpected phenotypes. By applying the FlowSOM clustering approach different clusters of B cells elicited by immunization with a tuberculosis vaccine antigen combined with the liposome-based adjuvant CAF01 have been characterized [57]. Employing a computational approach, it was possible to identify many plasmablast subsets and different germinal center B cell subtypes. The clustering approach, followed by a statistical analysis between groups immunized with or without the adjuvant component, has allowed us to identify a group of plasma cells as a specific biomarker of immunization with the adjuvanted-vaccine formulation [57]. Another clustering tool, FLOCK, was used for characterizing seventeen different B-cell subsets in human blood and to identify and quantify novel plasmablast subsets responding transiently to tetanus and other vaccinations (diphtheria toxoid, trivalent influenza vaccine 2009, H1N1 monovalent influenza vaccine, Hepatitis A, and Hepatitis B) [32].

Automated analysis can be particularly efficient also for identifying polyfunctional antigen-specific T cells elicited by vaccine administration or natural infection. Different computational tools, ranging from the Boolean combination gates, FlowSOM, or integrated approach combining targeted feature extraction (OpenCyto) with dimension reduction (t-SNE) have indeed been used to profile the polyfunctional activity of tuberculosis antigen-specific T cells and visualize treatment-specific differences between different vaccine formulations [106,107,108]. These studies demonstrate the importance of automated approaches to identify and visualize changes in very rare, multifunctional, antigen-specific T cells across different conditions, in flow cytometry datasets. 

## 8. Conclusions

Automated analysis of cytometric data has widely been demonstrated to efficiently achieve reproducible results compared to manual analysis, with the important advantages of eliminating the bias toward expected populations, the subjectivity in manual drawing of gates and in marker selection, and most importantly the possibility to identify unexpected cell populations. The potentiality of the automated analysis of cytometry data ranges from the improvement of the data quality in the pre-processing steps up to the unbiased, data-driven examination of complex dataset using a variety of algorithms based on different approaches. Automated tools such as clustering algorithms or dimensionality reduction techniques fully switch from the bi-variate to a multi-variate analysis, overcoming most of the drawbacks that affect classical manual analysis, which are still partially present in automated sequential gating and Boolean combination gates. Moreover, combined approaches using more than one algorithm can further improve the automated analysis [109]. The development of automated tools addresses many needs associated with high-dimensional datasets, and the awareness of their potential is now expanding from computer scientists to immunologists/biologists, as demonstrated by the rising numbers of scientific publication in fields such as oncology and immunology, reported in recent years. Nevertheless, this process is still at the beginning, and efforts aimed at encouraging interdisciplinary cooperation, simplifying the graphical user interface of the computational tools, and training the next generation of flow cytometry experts are necessary to further increase the application of automated analysis to complex cytometry data. The use of automated tools can significantly contribute to the interpretation of cytometric data in a more reliable and efficient way, and to improve the knowledge of cellular populations, their function and roles in physiological and pathological conditions. Cellular profiles obtained with automated analysis of complex flow cytometry datasets can be integrated through a systems biology approach with the molecular profile achieved with the *omic* technologies, such as genomics, transcriptomics, proteomics, and metabolomics, together with clinical readouts, for better understanding the behavior of the immune system in response to antigenic challenges, such as vaccination or infection.

## Figures and Tables

**Table 1:** Automated tools for the analysis of cytometric data. For each tool is reported the function, the statistical platform in which they are available, and a brief description of the main function.

Function	Software	Availability	Description	Reference
Pre-processing	FlowCore	R, Bioconductor	Import, compensate and transform FCS files in R environment	[9]
	FlowStats	R, Bioconductor	Collection of algorithms to analyze flow cytometry data, including correction of batch effect	[10]
	FlowClean	R, Bioconductor FlowJo plugin	Quality control of data set based on compositional analysis	[11]
	FlowAI	R, Bioconductor FlowJo plugin	Quality control of data set based on flow rate, signal acquisition and dynamic range	[12]
	CATALYST	R, Bioconductor	Collection of algorithms to pre-process cytometric data and to perform data analysis (with FlowSOM clustering and dimensionality reduction)	[13]
	CytoNorm	R	Normalized batch effect using control sample and clustering algorithm	[14]
Automated sequential gating	FlowDensity	R, Bioconductor	Provides tools for automated 1-D and 2-D sequential gating	[15]
	OpenCyto	R, Bioconductor	Facilitates automated 1-D and 2-D gating methods in sequential way to mimic the manual gating	[16]
	AutoGate	Standalone software	Performs 2-D sequential gating to obviate the need to draw arbitrary gates to define the subsets in a gating	[17]
	cytometree	R	The algorithm relies on the construction of a binary tree, the nodes of which represents cellular populations	[18]
	EPP	Standalone software	AutoGate extension. Algorithm that detects the best 2-D gating strategy to identify cellular populations	[19]
Boolean combination gates	flowType	R, Bioconductor	Phenotyping cytometric using multi-dimensional expansion of 1-D partitions	[20]
	FloReMi	R	Starting from flowType results identifies the populations that best correlates with an external outcome	[21]
	RchyOptimyx	R, Bioconductor	Starting from flowType results, constructs a hierarchy of cells selecting the most informative phenotypes for biomarker detection	[22]
Clustering	FlowMeans	R, Bioconductor FlowJo plugin	Automated gating tool based on K-means algorithm	[23]
	SPADE	R, Matlab, Cytobank, FlowJo plugin	Clustering method based combining density-based sampling with hierarchical clustering	[24]
	HDPGMM	Python	Clustering based on hierarchical modeling extensions to the Dirichlet Process Gaussian Mixture Model	[25]
	Citrus	Cytobank, R	Identifies cell populations with hierarchical clustering and make prediction with regression model	[26]
	FlowSOM	R, Bioconductor FlowJo plugin, Cytobank	Clustering method combining SOM and hierarchical clustering	[27]
	X-shift	Standalone software, FlowJo plugin	Clustering based on kNN density estimation and cluster merging according Mahalanobis distances	[28]
	flowClust	R, Bioconductor	Model-based clustering using a t-mixture model	[29]
	immunoClust	R, Bioconductor	Model-based clustering on individual samples. Includes an additional step to map cluster between samples	[30]
	SWIFT	Matlab	Clustering method based on splitting and merging of Gaussian mixture models	[31]
	FLOCK	C, Immport	Automated method partitioning of each dimension into bins, followed by merging of dense regions, and density-based clustering	[32]
	flowPeaks	R, Bioconductor	Clustering method combining density-based clustering and K-means	[33]
	ClusterX	R	Fast clustering by automatic search and find of density peaks	[34]
	PhenoGraph	Matlab, Python	Cells are visualized in a graph structure and connected with weighted edge based on neighbor shared by cell. Graph is then partitioned in group of cells sharing similar phenotypes	[35]
Dimensionality reduction	t-SNE	FlowJo plugin	Performs t-SNE in FlowJo, allowing to manually gate region in dimensionality reduced space to compare cell frequency across samples	[36]
	ACCENSE	Standalone software	Performs dimensionality reduction with t-SNE algorithm, followed by clustering of dimensionality reduced events with K-means or DBSCAN algorithms	[37]
	Rtsne	R	Performs t-SNE dimensionality reduction in R environment	[36]
	viSNE	Cytobank, Matlab	Visualization tool based on implementation of t-SNE algorithm	[38]
	EmbedSOM	R, Bioconductor FlowJo plugin	Dimensionality reduction technique based on SOM	[39]
	UMAP	R, Python, FlowJo plugin	Dimensionality reduction technique based on Uniform Manifold Approximation and Projection (UMAP)	[40]
	Destiny	R, Bioconductor	Performs dimensionality reduction with diffusion map	[41]
	Fit-SNE	R, Matlab, Python, FlowJo plugin	Tool to perform dimensionality reduction using Fast Fourier Transform-accelerated Interpolation-based t-SNE	[42]
Trajectory inference	Wanderlust	Matlab	Trajectory inference method based on kNN graph: Developed to identify linear transitions	[43]
	Wishbone	Matlab, Python	Evolution of Wanderlust, it can identify bifurcation in the trajectories	[44]
	Monocle	R, Bioconductor	Identification of bifurcated trajectory based on MST	[45]
	PHATE	Matlab, Python	Identification of trajectory preserving continual progressions, branches and clusters	[46]

R, package or code working on R; Bioconductor, R package available on Bioconductor repository [47]; Python, code or library written in Python language; Matlab, code or software based on Matlab language; C, code based on C programming language; FlowJo plugin, downloadable tools to expand FlowJo functionality [48]; Cytobank, online platform for single-cell analysis [49]; ImmPort, immunology database and analysis portal [50].

## Supplementary Materials

The following are available online at https://www.mdpi.com/2076-393X/8/1/138/s1, Figure S1: Comparison between Boolean combination of 1D gates and 2D gates.
