# Supplementary material for: From Bivariate to Multivariate Analysis of Cytometric Data: Overview of Computational Methods and Their Application in Vaccination Studies
Source: Vaccines (Basel). 2020 Mar 20;8(1):138. doi: 10.3390/vaccines8010138 (PMC7157606; doi:10.3390/vaccines8010138)
Supplement: Supplementary file 1 [file vaccines-08-00138-s001.pdf]

**Supplementary Materials**

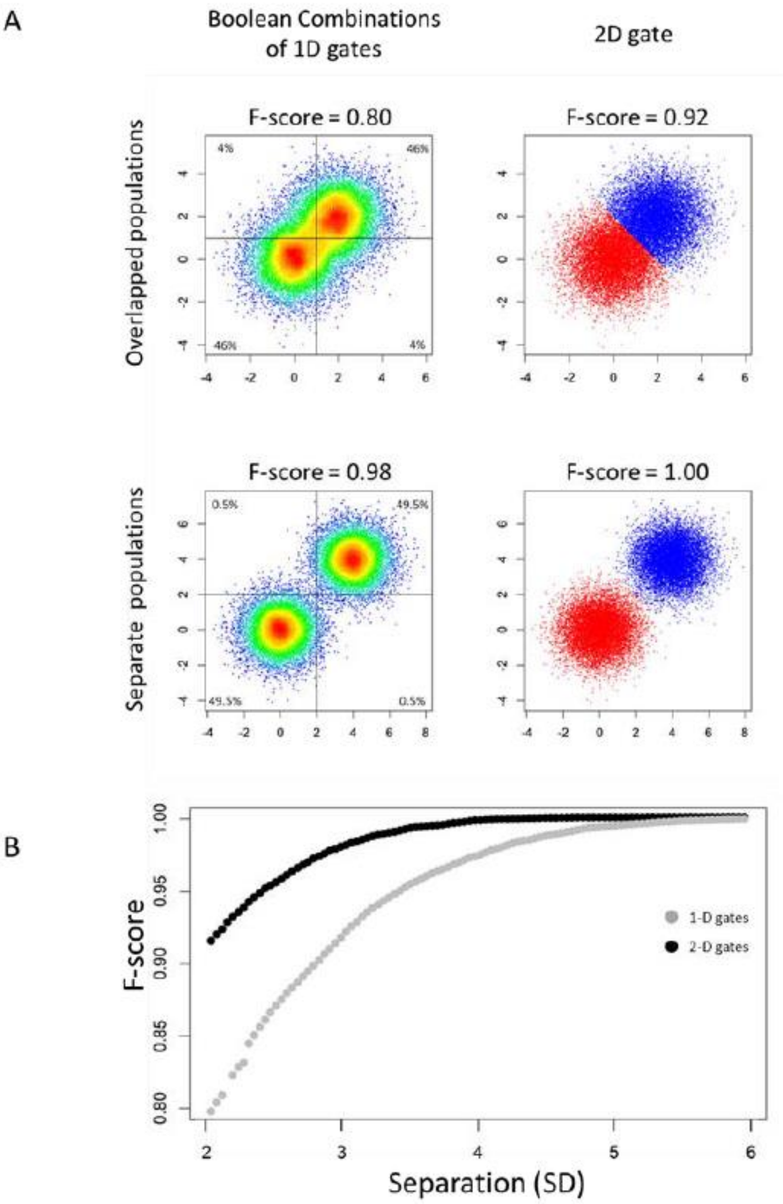


**Figure S1.** Comparison between Boolean combination of 1D gates and 2D gates. A bivariate dataset was generated as sum of two Gaussian distribution (standard deviation = 1 in both populations) in order to create two equally abundant populations: a double negative and a double positive. A Boolean combination of 1D gates identified with FlowDensity and a clustering algorithm (k-means) were used to analyze the bivariate dataset. FlowDensity and k-means were used to mimic manual 1D and 2D gates, respectively, in order to automatically analyze dataset with different overlap of two populations. The F-score is used to assess the quality of the gating. It is the harmonic mean of precision and recall and ranges between 0 and 1, with 1 indicating a perfect match between the predicted population and the reference. (**A**) Overlapped populations and well separate populations were analyzed and F-scores were reported. Boolean combinations gate failed to discriminate overlapped populations and identified artifact partitions (single positive populations). 2D gate gave better results even with overlapped populations. (**B**) The analysis was repeated with different separation between the two populations. F-scores (Y-axis) for each analysis were reported in blue for 2D gates and in red

for Boolean combination of 1D gates. X-axis represents the distance between the populations (in standard deviation, from 2 to 6).
